# Technology-Supported Guidance Models to Stimulate Nursing Students’ Self-Efficacy in Clinical Practice: Scoping Review

**DOI:** 10.2196/54443

**Published:** 2024-03-08

**Authors:** Paula Bresolin, Simen A Steindal, Hanne Maria Bingen, Jaroslav Zlamal, Jussara Gue Martini, Eline Kaupang Petersen, Andréa Aparecida Gonçalves Nes

**Affiliations:** 1 Federal University of Santa Catarina Santa Catarina Brazil; 2 VID Specialized University Oslo Norway; 3 Lovisenberg Diacinal University College Oslo Norway; 4 Ethics of Care Faculty of Health Sciences University of Stavanger Stavanger Norway

**Keywords:** clinical practice, guidance model, nursing education, review, self-efficacy, technology, mobile phone, nurse, nurses, nursing, education, allied health, synthesis, review methods, review methodology, search, searches, searching, scoping, MEDLINE, CINAHL, technology enhanced, Technology Enhanced Learning, digital health, guidance, model, models, practical, student, students

## Abstract

**Background:**

In nursing education, bridging the gap between theoretical knowledge and practical skills is crucial for developing competence in clinical practice. Nursing students encounter challenges in acquiring these essential skills, making self-efficacy a critical component in their professional development. Self-efficacy pertains to individual’s belief in their ability to perform tasks and overcome challenges, with significant implications for clinical skills acquisition and academic success. Previous research has underscored the strong link between nursing students’ self-efficacy and their clinical competence. Technology has emerged as a promising tool to enhance self-efficacy by enabling personalized learning experiences and in-depth discussions. However, there is a need for a comprehensive literature review to assess the existing body of knowledge and identify research gaps.

**Objective:**

The aim of this study is to systematically map and identify gaps in published studies on the use of technology-supported guidance models to stimulate nursing students’ self-efficacy in clinical practice.

**Methods:**

This scoping review followed the framework of Arksey and O’Malley and was reported according to the Preferred Reporting Items for Systematic Reviews and Meta-Analyses for Scoping Reviews (PRISMA-ScR). A systematic, comprehensive literature search was conducted in ERIC, CINAHL, MEDLINE, Embase, PsycINFO, and Web of Science for studies published between January 2011 and April 2023. The reference lists of the included papers were manually searched to identify additional studies. Pairs of authors screened the papers, assessed eligibility, and extracted the data. The data were thematically organized.

**Results:**

A total of 8 studies were included and four thematic groups were identified: (1) technological solutions for learning support, (2) learning focus in clinical practice, (3) teaching strategies and theoretical approaches for self-efficacy, and (4) assessment of self-efficacy and complementary outcomes.

**Conclusions:**

Various technological solutions were adopted in the guidance models to stimulate the self-efficacy of nursing students in clinical practice, leading to positive findings. A total of 7 out of 8 studies presented results that were not statistically significant, highlighting the need for further refinement of the applied interventions. Nurse educators play a pivotal role in applying learning strategies and theoretical approaches to enhance nursing students’ self-efficacy, but the contributions of nurse preceptors and peers should not be overlooked. Future studies should consider involving users in the intervention process and using validated instruments tailored to the studies’ intervention objectives, ensuring relevance and enabling comparisons across studies.

## Introduction

Nursing students need to acquire both theoretical knowledge and practical skills during their education. Clinical practice is essential for their achievement of competence in communication, teaching, examinations, treatments, management, cooperation, professional approach, and the nursing process [1], yet nursing students experience several challenges in acquiring such competence and skills in clinical practice [2].

Self-efficacy theory has drawn great attention in the health care setting of nursing education and clinical practice. The concept of self-efficacy refers to people’s belief in their capability to perform a task or handle a challenging situation [3] and has been used to bridge the theory-practice gap and promote clinical skills acquisition, critical thinking, and general academic success [4-6], so it is important to find ways to foster self-efficacy among nursing students during their clinical education. Effective clinical training of nursing students can enhance self-efficacy, which is a key component of acting independently and competently in the nursing profession [7,8]. Furthermore, nursing students’ clinical performance, course completion, and motivation for achievement are closely linked to their perceived self-efficacy [8,9].

Previous research has consistently demonstrated a strong association between nursing students’ clinical competence and their overall self-efficacy levels [10,11]. Nursing students with a high degree of self-efficacy tend to exhibit advanced problem-solving skills [12] and demonstrate a strong capacity for self-regulated learning [13], which are critical attributes in their professional training and development. However, nursing students’ learning performance and self-efficacy can be significantly impacted by a lack of adequate support to master the complex knowledge and skills required in nursing [14]. Robb [4] found that nursing students with low self-efficacy required emotional and academic support and suggests that nurse educators should be attentive to the strategies millennial students use to acquire information and should provide constructive feedback on student performance. This strategic approach is equally pertinent for Generation Z students, recognized as digital natives, currently undergoing higher education. As they present specific challenges for nurse educators, adapting teaching-learning design strategies and approaches also becomes imperative [15].

The advancement of technology has opened new possibilities for supporting nursing students’ knowledge, competence, and skills acquisition in clinical practice [16]. Technology has great potential to improve nursing education by enabling personalized interaction and in-depth discussions of learning topics [16] and by enhancing self-efficacy [17]. The use of customized technological tools in nursing education remains somewhat limited [18]. Earlier systematic reviews have demonstrated opposing results regarding the effectiveness of technology-supported interventions in nursing education [19,20]. The review by Lee et al [19] found that smartphone-based apps could promote nursing students’ learning motivation and satisfaction but not their clinical skills and knowledge. In contrast, the review by Kim and Park [20] demonstrated that mobile-based learning could effectively support nursing students’ acquisition of knowledge and skills both in and outside of clinical practice settings [20]. Traditionally, clinical practice has played a crucial role in nursing education, organized by guidance models. These models consist of procedures, meetings, and collaboration, aiming to facilitate the development of nursing students’ competencies in clinical practice through cooperation between health care and educational institutions [21]. The concept of a technology-supported guidance model in nursing education entails integrating tools, theories, and technological resources to improve guidance and support throughout students’ educational journey. The implementation of these technological models, including online platforms, virtual simulations, and digital resources, seeks to enhance the effectiveness and interactivity of nursing education, tailoring it to the users’ individual needs [22]. Technology-supported guidance models represent an evolution in teaching methods, incorporating technological elements to improve learning quality and meet the demands of the current educational landscape, aligned with clinical practice expectations. Such models are designed to integrate technological tools into guidance systems, thereby enhancing knowledge and improving students’ attitudes and learning outcomes [22].

Given the crucial role of self-efficacy in nursing students’ learning process in clinical practice and the potential of technology to optimize the stimulation of self-efficacy, a broad literature review is needed to provide an overview of the published studies on this phenomenon and identify possible research gaps. Our initial literature searches identified only 2 reviews: a systematic mixed studies review synthesized existing evidence on technology-supported guidance models in nursing education, focusing on the development of critical thinking in nursing students in clinical practice [23], and an integrative review evaluated studies on the collaborative use of mobile devices by nursing students and nurse educators during clinical practice but did not investigate the impact on self-efficacy [24]. We were not able to identify previous scoping reviews addressing self-efficacy in the context of technology-supported guidance models in clinical practice in nursing education. Therefore, this scoping review aimed to systematically map and identify gaps in published studies on the use of technology-supported guidance models to stimulate nursing students’ self-efficacy in clinical practice.

## Methods

### Overview

This scoping review used Arksey and O’Malley [25] five-stage framework: (1) identification of the research question; (2) identification of relevant studies; (3) selection of studies; (4) mapping the data; and (5) gathering, summarizing, and reporting the results. The reporting of the review was guided by the Preferred Reporting Items for Systematic Reviews and Meta-Analyses for Scoping Reviews (PRISMA-ScR) [26]. The review’s protocol was not registered or published.

### Research Question

What is known about the use of technology-supported guidance models to stimulate nursing students’ self-efficacy in clinical practice?

### Identification of Relevant Literature

A systematic search was conducted on December 13, 2021, and was updated on April 21, 2023, in the following databases: MEDLINE All (Ovid), PsycINFO (Ovid), Embase (Ovid), ERIC (EBSCOhost), CINAHL (EBSCOhost), and Web of Science Core Collection. The search strategy consisted of three main topics: (1) self-efficacy, (2) technology, and (3) nursing students. Based on these 3 topics, we chose search terms using Medical Subject Headings terms and text words. The search strategy was built in MEDLINE by a research librarian, peer reviewed by a second research librarian, and then adapted to the other databases (Multimedia Appendix 1). Moreover, we hand-searched the reference lists of the included papers to assess whether any of the studies mentioned in those references were pertinent to our review. Furthermore, we conducted forward citation searching using the Google Scholar platform to identify relevant studies that had cited the included papers.

### Selection of the Studies

The research librarian exported the identified citations to EndNote (Clarivate) to remove duplicates [27]. Subsequently, the citations were exported to Rayyan (Rayyan Systems Inc) [28] for storage, organization, and blinding of the study selection process. Based on the eligibility criteria (Textbox 1) [29], PB and AAGN independently conducted a pilot test of 10% (380/3804) of the citations to screen titles and abstracts, and the eligibility criteria were not modified. Pairs of authors independently screened titles and abstracts to evaluate whether full-text studies met the eligibility criteria (PB+AAGN, Fernando Riegel+JGM, and SAS+JZ in the first search round and PB+JGM and SAS+HMB in the update search round). When doubt arose about a full-text study’s inclusion, a third author independently evaluated the full-text study. The decision was based on a negotiated consensus, and the reasons for excluding full-text studies were recorded.

**Eligibility criteria according to the Sample, Phenomenon of Interest, Design, Evaluation, and Research type (SPIDER) framework.**

**Inclusion criteria**

- Sample (S): studies including undergraduate nursing students.
- Phenomenon of interest (PI): use of technology to support guidance in clinical practice to stimulate self-efficacy or similar concepts in an educational institutional context.
- Design (D): studies with qualitative, quantitative, or mixed methods published in peer-reviewed journals from January 2011 to April 21, 2023 (based on our preliminary research, we concluded that the field of Technology-Supported Guidance Models in clinical practice in nursing education is a relatively new research area, and the likelihood of finding studies published in this area before 2011 was low).
- Evaluation (E): undergraduate nursing students’ self-efficacy in using technology for stimulating self-efficacy or similar concepts.
- Research type (R): studies of any research type published in English, Portuguese, Spanish, Norwegian, Danish, or Swedish published in peer-reviewed journals.

**Exclusion criteria**

- Sample (S): studies including health care students other than undergraduate nursing students.
- Phenomenon of interest (PI): educational guidance supported by technology to stimulate self-efficacy unrelated to clinical practice or an educational institution context.
- Design (D): studies published before January 2011 or after April 21, 2023.
- Evaluation (E): the self-efficacy of other health care students’ or professionals or nurse educators when it comes to using technology to stimulate self-efficacy.
- Research type (R): non–peer-reviewed studies, any type of review, case study, case report, clinical guideline, master’s or PhD thesis, conference proceedings, abstracts, letters, comments, discussion editorials, books, or book chapters.

### Mapping the Data

The same pairs of authors that selected the studies extracted data from the included studies. One extracted the data, maintaining the wording and terminology of the studies, and the other checked data accuracy against the studies using a standardized data charting form that included the following information as recommended by the Joanna Briggs Institute [30]: authors, year, country, study objective, population and sample size, research focus or technological models, design, outcomes measures (related to self-efficacy), and findings.

### Critical Appraisal

In line with the framework by Arksey and O’Malley [25], a critical appraisal of the methodological quality or risk of bias of the included studies was not performed.

### Grouping, Summarizing, and Reporting the Results

PB and AAGN used an inductive approach to analyze and thematically organize the data from the included studies [25]. The data were extracted from the studies’ findings sections and were read several times to identify patterns of similarities and differences across the studies related to our research question. These patterns were organized into thematic groups using a low level of abstraction. Next, the preliminary thematic groups were discussed with the rest of the research team and a consensus was achieved [31-33].

## Results

### Overview

**Figure 1:**
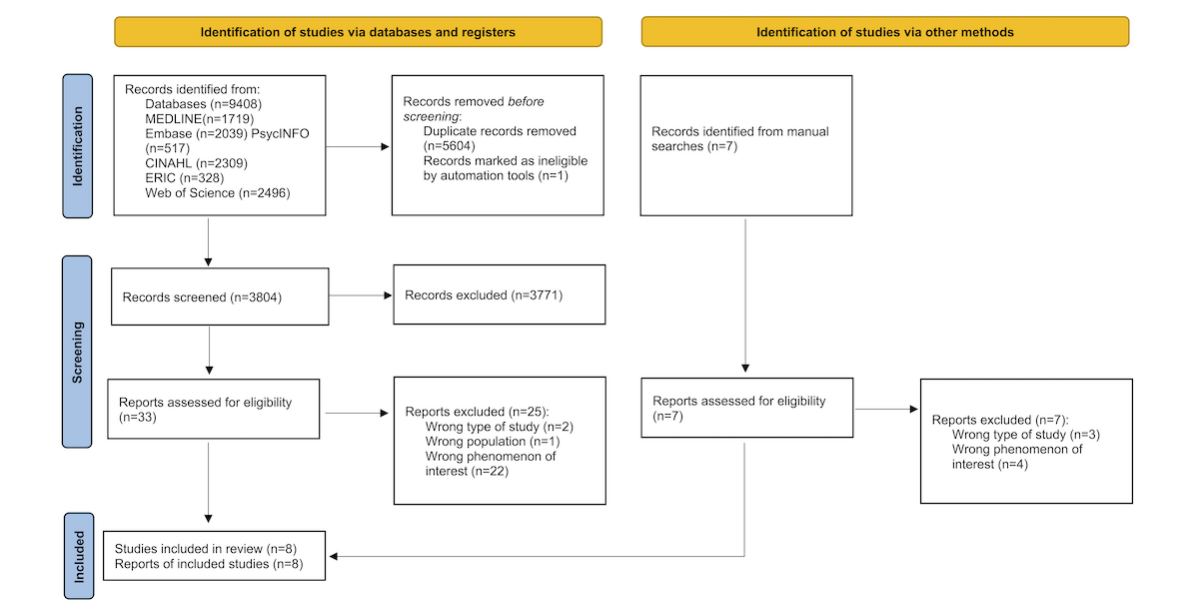
PRISMA (Preferred Reporting Items for Systematic Reviews and Meta-Analyses) flow diagram.

The database search identified 9408 records, of which 5604 were duplicates, so we screened the titles and abstracts of 3804 records. A total of 33 studies were evaluated for eligibility and 8 studies described in 8 publications were included. We did not find any relevant additional studies through hand searches of the reference lists or forward citations of the included studies. Figure 1 provides an overview of the study selection process and the reasons for the exclusion of full-text reports.

### Study Characteristics

The included studies were conducted in Taiwan (n=2) [34,35], South Korea (n=2) [36,37], China (n=2) [36,38], Norway (n=1) [39], and Finland (n=1) [40]. In all, 5 studies used a quantitative method and had an experimental design [37-41], and 3 studies used multiple methods [34-36].

The sample size of the studies ranged from 36 to 171 participants, the majority age range (721/770, 93.6%) across the studies was 20-30 years, and most of the participants were female (508/564, 90%). A total of 3 studies did not report the sex of the participants [34,35,37]. In most of the studies (7/8, 88%), the clinical practice was performed in a hospital [34,36-41], whereas 1 study’s clinical practice was performed in home care [35]. Table 1 provides a detailed overview of the studies’ characteristics and Multimedia Appendix 2 [34-41] provides a description of interventions, duration, and frequency for each study included.

**Table 1:** Characteristics of the included studies.

Author, year, and country	Study objectives	Population and sample size	Research focus or technological models	Design	Outcomes related to self-efficacy	Findings
Chang et al, 2022 [34], Taiwan	To enable students to learn and think deeply by interacting with a chatbot in the context of handling obstetric vaccine cases	N=36 EG^a^: n=18 CG^b^: n=18 Age: mean=21 years Sex: NR^c^	Nursing procedures or chatbot applying natural language processing	Nonrandomized controlled trial with quantitative and qualitative approach	Self-efficacy questionnaire based on Pintrich et al [42]. Individual interviews to investigate students’ perceived self-efficacy	Qualitative and quantitative data indicate that applying the mobile chatbot as a learning strategy enhanced nursing students’ self-efficacy
Egilsdottir et al, 2023 [39], Norway	To explore changes in nursing competence, factors associated with changes after clinical rotations, and whether an SMLT^d^ supports changes in the confident use of B-PAS^e^	N=171 Age: The median is between 21-25 years Sex: n=154 female	B-PAS or SMLT	Quantitative cohort study	Study-specific questionnaire to investigate students’ confidence in performing physical assessments	After the clinical rotation, both student groups reported changes in the confidence in performing B-PAS, with statistically significant moderate or large changes in all areas. Confidence in performing B-PAS, the usefulness of the SMLT, and a higher nursing competence at the start of clinical rotation were positively associated with overall nursing competence
Kim and Suh, 2018 [41], South Korea	To evaluate the effect on nursing students of an ICNS^f^ mobile app	N=66 EG: n=34 Age: mean=22.6 years Sex: n=31 female CG: n=32 Age: mean=22.9 years Sex: n=29 female	Simulation of nursing procedures or ICNS app	Randomized controlled trial	SECP^g^ instrument	The ICNS app enhanced students’ knowledge, self-efficacy, and nursing skills performance. The EG showed significantly more improved self-efficacy from before to after the intervention than the CG
Lee and Park, 2018 [37], South Korea	To examine the effect of flipped learning compared to traditional learning in a surgical nursing practicum	N=102 EG: n=51 Age: mean=22.5 years Sex: NR CG: n=51 Age: mean=22.5 years Sex: NR	Clinical practice in surgical nursing or flipped learning with e-learning content with smart learning tool	Two-arm, parallel, stratified group randomized trial	SECP instrument	Both groups showed improvement on all subscales of the SECP in the posttest, but no statistically significant differences were found between the group
Strandell-Laine et al, 2018 [40], Finland	To evaluate the effectiveness of a mobile cooperation intervention in improving students’ competence and self-efficacy and the quality of the CLE^h^	N=102 EG: n=52 Age: mean=22.9 years Sex: n=49 female CG: n=50 Age: mean=23 years Sex: n=45 female	Clinical practice or mobile app	Randomized controlled trial	SECP instrument	The results of overall competence, self-efficacy, and overall satisfaction with the CLE showed no significant differences between the groups
Wang et al, 2022 [38], China	To examine the effects of a mobile phone–based psychological intervention program on stress, anxiety, and self-efficacy among undergraduate nursing students during clinical practice	N=114 EG: n=57 Age: mean=22.9 years Sex: n=50 female CG: n=57 Age: mean=22.2 years Sex: n=47 female	Psychological intervention or mobile phone–based	Randomized controlled trial	General Self-Efficacy Scale	More significant improvements in stress, anxiety, and self-efficacy as well as more significant improvement in group-interaction time were observed in the EG than in the CG
Wang et al, 2023 [36], China	To develop and evaluate the effectiveness of an online 5-week professional identity program among nursing students in clinical internship practice during COVID-19 restrictions	N=111 EG: 56 Age: mean=21.3 years Sex: n=53 female CG: 55 Age: mean=21.3 years Sex: n=50 female	Professional identity or online program	Two-armed randomized controlled trial with quantitative and qualitative approach	Professional self-efficacy questionnaire for nursing students	For professional self-efficacy, the group effect, time effect, and group-by-time effect were not significant except for 1 factor related to the capacity for information collection and planning. Students stated that the program enhanced their professional belief, and they felt less stressed in adapting to the stressful atmosphere. The facilitator supported the participants’ experiences of internal self-motivation that led to active participation in the program. Building mutual trust and familiarity was essential for the group dynamic
Wu and Sung, 2014 [35], Taiwan	To assess the advantages of mobile devices and cloud learning in a public health practice course using Google+ as the learning platform and integrating various application tools	N=68 EG: n=32 Age: NR Sex: NR CG: n=36 Age: NR Sex: NR	Clinical practice in public health or Google+ as a learning management system	Nonrandomized pilot study with quantitative and qualitative approach	Computer self-efficacy instrument designed by Compeau and Higgins [43]	Most students had past computer experience and often searched for information on the internet. They were confident in computer use and displayed high self-efficacy. The analysis of learning effectiveness showed that students using Google+ had greater learning effectiveness than did those adopting traditional learning

^a^EG: experimental group.

^b^CG: control group.

^c^NR: not reported.

^d^SMLT: suite of mobile learning tools.

^e^B-PAS: basic physical assessment skills.

^f^ICNS: interactive clinical nursing skills.

^g^SECP: Self-Efficacy in Clinical Performance.

^h^CLE: clinical learning environment.

### Thematic Groups

To answer the research question, the results were organized into four thematic groups: (1) technological solutions for learning support, (2) learning focus in clinical practice, (3) teaching strategies and theoretical approaches for self-efficacy, and (4) assessment of self-efficacy and complementary outcomes. Textbox 2 provides an overview of the content covered within the thematic groups.

**Content covered within the thematic groups.**

**Technological solutions for learning support**

- Chatbot with artificial intelligence [34]
- Digital platform [35,37]
- Mobile app with simulation [39,41] and without simulation [40]
- Mobile phone [38]
- Online chat [36]

**Learning focus in clinical practice**

- Nursing procedures: vaccine [34], vital signs, intravenous injection, gastric lavage, endotracheal suction [41], and physical assessment skills [39]
- Specialized nursing area: surgical nursing [37], home care [35], and management and communication [40]
- Students’ professional identity [36]
- Students’ mental health [38]

**Teaching strategies and theoretical approaches for self-efficacy**

- Attention, relevance, confidence, and satisfaction theory [41]
- Flipped classroom [37]
- Fundamentals of Care framework [39]
- Nurse educators’ feedback on nurse students’ learning activities [35,38,40]
- Tajfel’s social identity theory and career self-efficacy theory [36]

**Assessment of self-efficacy and complementary outcomes**

- Computer self-efficacy, experience, anxiety, and system satisfaction and interview [35]
- Confidence in performing basic physical assessment skills and nurse professional competence [39]
- General self-efficacy, learning situation, and interview [34]
- General self-efficacy, stress, and anxiety [38]
- Professional self-efficacy, professional identity, depression, anxiety, stress, and interview [36]
- Self-efficacy in clinical performance (SECP), nursing skills performance, and knowledge [41]
- SECP, quality of the clinical learning environment, and nurse competence [40]
- SECP, self-leadership, and social problems [37]

### Technological Solutions for Learning Support

Various technological solutions to support the development of self-efficacy were identified across the included studies, such as a chatbot with artificial intelligence [34], online chat [36], a mobile app with simulation [39,41] and without it [40], a mobile phone [38], and a digital platform [35,37]. The technological tools required internet access to function for the intended purpose. In 7 studies [34-36,38-41], the participants had access to the technological solution on their smartphones anywhere and at any time. The participants used the technical solutions to perform learning activities [34,35,37,39-41] to interact with colleagues [34-36] and to communicate with nurse educators [35,38,40]. In 1 study, the technological solution (with e-learning content) was used as a preparatory learning activity before clinical practice [37].

### Learning Focus in Clinical Practice

The included studies focused on specific learning situations in clinical practice, such as nursing procedures [34,39,41] and nursing areas [35,37,40]. Regarding nursing procedures, 1 study provided educational knowledge about infectious diseases and vaccine administration [34], while the other studies included learning situations about vital signs, intravenous injection, gastric lavage, endotracheal suction [41], and physical assessment skills [39]. Regarding nursing areas, the research focus was surgical nursing [37], management and communication [40], and home care [35]. One study investigated students’ mental health [38] and another investigated students’ professional identity [36].

### Teaching Strategies and Theoretical Approaches for Self-Efficacy

Nurse educators were the main facilitators of the technology-supported guidance models. A total of 4 studies applied various teaching strategies, such as educators’ feedback on nursing students’ learning activities [35,36,38,40] and a flipped classroom [37]. Three of the studies used different theoretical approaches, such as attention, relevance, confidence, satisfaction (ARCS) theory [41], Tajfel’s social identity theory (SIT) and career self-efficacy theory (CSET) [36], and the Fundamentals of Care (FoC) framework [39]. One study did not apply teaching strategies or theoretical approaches [34].

Strandell-Laine et al [40] and Wu and Sung [35] designed an app to support clinical learning by stimulating communication between nursing students and nurse educators. In the intervention by Strandell-Laine et al [40], the learning content included the schedule of clinical practice, learning objectives, a learning diary, and midterm and final evaluations. In the study by Wu and Sung [35], students accessed information, uploaded data, posed questions, and discussed the learning situations with nurse educators. In both studies, the intervention content and nursing students’ clinical practice experience were the basis of feedback elaboration delivered by the educators. Nursing students also received feedback from nurse educators in Wang et al [38] study, but the focus was on psychological support. The intervention was delivered in three modules: (1) support (the participants were asked to write a paragraph describing their “happy experiences” during their clinical practice sessions); (2) education (2 clinical educators provided weekly lectures on topics to improve clinical and communication skills, find happiness in daily life, build confidence when caring for patients, manage stress and pressure, perform self-care while caring for others, and build social support); and (3) reflection (participants were encouraged to describe stressful situations during their clinical practice to their clinical educators, and the educators helped the participants analyze the situations and provided tailored advice for handling similar situations in the future). In the study by Lee and Park [37], the flipped classroom was used as a teaching strategy, including instructor guidance before clinical training, on-site instruction during clinical practice, and a case conference after the end of clinical practice.

The study by Chang et al [34] used an artificial intelligence–based app developed with natural language processing to encourage nursing students to ask questions or use a pop-up menu to search for needed information in addition to discussing medical issues with their peers and with the chatbot. Kim and Suh [41] used an app flowchart based on the ARCS theory with four phases: (1) the attention phase stimulated the participants’ motivation to learn; (2) the relevance phase helped the participants to think about which items they should prepare for each nursing skill and devise a care plan for the patients and themselves; (3) confidence phase; and (4) satisfaction phase, the participants learned interactively by answering messages and quizzes that popped up on their mobile screen. Egilsdottir et al [39] used the FoC framework divided into three main areas: (1) the nurse-patient relationship, (2) integration of care, and (3) contextual factors The study used the basic physical assessment skills (B-PAS) to measure students’ performance and used the FoC framework to assess the student (nurse)–patient relationship. Wang et al [36] based their training program on the combined SIT and CSET. The program was designed on the basis of SIT’s 3 phases of how social identity is built and modified, including social categorization, social comparison, and positive distinctiveness; the intervention elements embedded in the 3 phases were derived from the CSET, comprising direct experience, substituted experience, physio-psychological condition, and social persuasion.

### Assessment of Self-Efficacy and Complementary Outcomes

Three studies [37,40,41] measured self-efficacy using the self-efficacy in clinical performance (SECP) instrument [44], which comprises 37 self-assessed items on an 11-point Likert scale in five domains: (1) assessment, (2) diagnosis, (3) planning, (4) implementation, and (5) evaluation.

Two studies [34,38] measured general self-efficacy with 2 different instruments. Chang et al [34] used a self-efficacy instrument constructed by Pintrich et al [42] that includes 8 self-assessed items with 5-point Likert scales. Wang et al [38] used a self-efficacy scale developed by Jerusalem and Schwarzer [45] with 10 items on a 4-point Likert scale in four domains: (1) strategic, (2) contingency, (3) motivational, and (4) executive effectiveness.

Wang et al [36] measured nursing students’ professional self-efficacy with a 27-item questionnaire on a 5-point Likert scale including six factors: (1) professional attitude and belief, (2) problem-solving ability, (3) professional information collection and professional planning capacity, (4) professional cognition, (5) professional value, and (6) professional choice.

Egilsdottir et al [39] created a questionnaire with 13 items using a 7-point Likert scale to map nursing students’ perceived confidence related to the examination techniques in B-PAS, which are inspection, palpation, percussion, and auscultation. The questionnaire items were formulated in line with Bandura’s [46] description of self-efficacy.

Wu and Sung [35] revised and applied a computer self-efficacy questionnaire designed by Compeau and Higgins [43] with 24 items on a 5-point Likert scale in three dimensions: (1) computer use experience, (2) computer self-efficacy, and (3) computer anxiety.

In all these self-efficacy instruments, higher scores indicate greater self-efficacy. All the studies measured self-efficacy along with other outcomes, such as basic knowledge [34,41], nursing skills performance [39,41], self-leadership and social problems [37], and professional competence [39,40]. Three studies measured anxiety [35,36,38], 2 measured stress [36,38], 1 measured professional identity [36], and another measured system satisfaction [35]. Furthermore, 3 studies [34-36] conducted interviews to gather qualitative data, exploring participants’ perceptions of their feelings, experiences, influencing factors, their understanding of technology’s impact on learning experiences in nursing education, and suggestions for improving the intervention.

In all the studies, the self-efficacy scales and other instruments showed improvement in the posttest within the groups. However, 1 showed statistically significant differences between the experimental groups and the control groups [34].

## Discussion

### Principal Findings

This scoping review aimed to systematically map and identify gaps in published studies on the use of technology-supported guidance models to stimulate nursing students’ self-efficacy in clinical practice. The database search identified 3804 citations, but only 33 studies were assessed for eligibility, of which 8 were eligible for inclusion, which may indicate a research gap on the phenomenon of interest. Although the number of studies was small, the technological solutions were diverse, which was not surprising, as it aligns with the findings of a systematic mixed studies review that investigated technology-supported guidance models to stimulate critical thinking [23]. The use of technology to support clinical practice appears to be a relatively new research field, characterized by frequent experimentation. Technological advancement in nursing education has greatly increased, especially during the COVID-19 pandemic [47].

Our findings suggest that technological solutions were organized as technology-supported guidance models with a predefined set of nursing procedures or nursing areas. Nurse educators provided guidance with the support of technological solutions, stimulating nursing students’ active learning. This pedagogical approach diverges from traditional education and seeks to transcend teaching based on the unilateral transfer of content so as to stimulate creative, critical, and transformative practices [48]. Active learning is student centered and interactive and offers feedback that meets the student’s learning needs [49]. Although only 1 of the included studies [39] provided information on the development of the technological solutions, they seemed to be tailored to meet the individual nursing students’ needs for specific knowledge and competence. It would be valuable to ascertain whether the users of the other 7 included studies were actively engaged in the development process due to the potential positive impact of such involvement. Nes et al [50] underscore the significance of incorporating all stakeholders (ie, nursing students, nurse preceptors, and nurse educators) as users during the creation of a technology-supported guidance model. This approach is essential to guarantee that the technological solution aligns with the expected quality standards to meet users’ needs and achieve the anticipated educational outcomes in clinical practice.

Half (4/8, 50%) of the included studies applied teaching strategies without a theoretical approach in their technology-supported guidance models, which is in line with the findings of a previous review [23]. The lack of a theoretical approach may make it challenging to explain study findings [51]. Despite positive findings, only 1 of the included studies showed statistically significant effects regarding self-efficacy. Applying a theoretical approach in intervention studies seems to be associated with positive findings and large effect sizes [52]. Therefore, technology-supported guidance models that apply pedagogical theory intended to stimulate self-efficacy may have a better chance of success [53]. A nonsignificant effect in the included studies that used a theoretical approach may result from an insufficient sample size or insufficient duration of intervention. Despite the lack of statistical significance in most studies, the observed effects were consistently positive, aligning with findings in other studies using technological solutions [54-56]. Consequently, interventions integrating technology have the potential to contribute positively to student learning outcomes.

Furthermore, Linnenbrink and Pintrich [57] found evidence for a conceptual framework that demonstrated the connection between motivation, self-regulation, and academic learning and that these connections were not confined solely to the theoretical classroom context but also extended to clinical practice. Motivational factors and cognitive processes may interact in intricate ways to facilitate student learning [57,58].

Aligning with previous research [59,60], our findings underscore the significant role of nurse educators as the primary facilitators of technology-supported guidance models. Nurse educators share responsibility for fostering nursing students’ self-beliefs, as these self-beliefs can have positive or negative influences on their performance [61]. Educators and institutions are responsible for helping students develop their competence and confidence as they progress in their studies [62]. However, technological tools should not be incorporated into guidance models in isolation, as such incorporation also requires oversight, support, and mentorship from not only nurse educators but also nurse preceptors and peers optimizing the impact of technology on the educational experience [63].

Only 3 of the included studies used the SECP instrument to assess the self-efficacy of nursing students. Using the same instrument, such as the SECP, facilitates replication and enables comparisons across studies investigating similar outcomes [44,64,65]. By contrast, the use of different instruments to measure the same outcome makes it challenging to compare findings across studies, conduct meta-analyses, and establish standardized thresholds or reference points for specific outcomes [66].

Our findings show that the studies also incorporated other assessment tools. This could be because of the strong correlation between self-efficacy and factors such as motivation [67], satisfaction [63,68], academic achievement [58,69], and student persistence [70]. Furthermore, it is important to acknowledge that other outcomes could be essential in addressing the research questions posed by these studies.

### Strengths and Limitations

The strengths of our review are the acknowledged methodological framework for conducting a scoping review, the comprehensive database search, and the systematic process by which pairs of authors independently assessed eligibility and extracted data. Furthermore, the data were analyzed by 2 authors and discussed with the rest of the research team, facilitating credibility, dependability, and intersubjectivity.

We tried to include all possible synonyms of the concept of self-efficacy and similar concepts in our search strategy, but due to the multidimensional nature of self-efficacy, we may have overlooked some synonyms. Our review also had some language restrictions. Consequently, we may have been unable to identify some relevant studies. Another limitation may be that the review protocol was not published. However, the eligibility criteria and search strategy were determined before the study selection process was carried out.

### Conclusions

Diverse technological solutions were used in guidance models to stimulate nursing students’ self-efficacy in clinical practice. Even though these interventions showed positive outcomes, they were not statistically significant. This underscores the need for further refinement by tailoring technological tools to meet user needs, making stakeholder involvement essential, and implementing interventions that are developed on the basis of a theoretical approach, as well as applying teaching strategies with a theoretical approach. Although nurse educators are vital for students’ development, the contributions of nurse preceptors and peers should not be underestimated.

Our findings show that a variety of instruments are used to assess self-efficacy and that not all such instruments have been validated. Consequently, future studies should use validated instruments to ensure relevance and enable meaningful comparisons of self-efficacy across studies.

## Appendix

Multimedia Appendix 1 Search strategy used in MEDLINE.

Multimedia Appendix 2 The description of interventions for each study included.

Multimedia Appendix 3 PRISMA-ScR checklist.

## Abbreviations

ARCS: attention, relevance, confidence, satisfaction
B-PAS: basic physical assessment skills
CSET: career self-efficacy theory
FoC: Fundamentals of Care
PRISMA-ScR: Preferred Reporting Items for Systematic Reviews and Meta-Analyses for Scoping Reviews
SECP: self-efficacy in clinical performance
SIT: social identity theory
