# Supplementary material for: Technology-Supported Guidance Models to Stimulate Nursing Students’ Self-Efficacy in Clinical Practice: Scoping Review
Source: JMIR Nurs. 2024 Mar 8;7:e54443. doi: 10.2196/54443 (PMC10960213; doi:10.2196/54443)
Supplement: Multimedia Appendix 1 [file nursing_v7i1e54443_app1.docx]

| 1 | nursing students/ |
| --- | --- |
| 2 | nursing education/ |
| 3 | ((nurse or nursing or nurses or BSN or BN) adj4 (student* or educati* or undergrad* or under-grad* or bachelor* or baccalaur* or pre-registration or preregistration or school* or college* or universit*)).tw. |
| 4 | 1 or 2 or 3 |
| 5 | Videoconferencing/ |
| 6 | Wireless Technologies/ |
| 7 | telephone systems/ |
| 8 | exp mobile phones/ |
| 9 | text messaging/ |
| 10 | smartphone use/ |
| 11 | Computer Mediated Communication/ |
| 12 | telecommunications media/ |
| 13 | technology/ |
| 14 | mobile applications/ |
| 15 | computers/ |
| 16 | Mobile Devices/ |
| 17 | exp Microcomputers/ |
| 18 | Computer usage/ |
| 19 | Tablet Computers/ |
| 20 | computer assisted instruction/ |
| 21 | Internet/ |
| 22 | Internet Usage/ |
| 23 | teleconferencing/ |
| 24 | electronic learning/ |
| 25 | mobile learning/ |
| 26 | distance education/ |
| 27 | Websites/ |
| 28 | Mobile Technology/ |
| 29 | Electronic Communication/ |
| 30 | (phone* or phoning or telephon* or tele-phon* or cellphon* or cell-phon* or smartphon* or smart-phon* or mobile* or laptop* or lap-top*).tw. |
| 31 | ((portable or handheld or hand-held or pocket or personal) adj (computer* or pc or pcs or device*)).tw. |
| 32 | (pda or “personal digital assistant*” or “personal data assistant*” or “palm computer*” or “palm pc” or “palm pcs” or “palm pilot*” or palmtop* or palm-top* or microcomputer* or minicomputer* or tablet*1 or ipad*).tw. |
| 33 | (internet or app*1).tw. |
| 34 | (telecommunicat* or tele-communicat*).tw. |
| 35 | (communicat* adj3 (computer* or electronic* or web* or digital* or technolog* or online or video*)).tw. |
| 36 | (teleconferenc* or tele-conferenc* or "electronic conferenc*" or e-conferenc* or web-conferenc* or "digital conferenc*" or "online conferenc*" or video-conferenc* or videoconferenc* or webinar* or webcast* or web-cast*).tw. |
| 37 | (web-supported or web-based or web-assisted or web-enhanced or web-site* or website*).tw. |
| 38 | (computer-supported or computer-based or computer-assisted or computer-enhanced or technology-supported or technology-based or technology-assisted or technology-enhanced or video-supported or video-based or video-assisted or video-enhanced).tw. |
| 39 | (etutor* or e-tutor* or e-learn* or elearn* or “distance education” or “distance learning”).tw. |
| 40 | (“text messag*” or “short messag*” or “instant messag*” or texting or sms or “synchronous communication*”).tw. |
| 41 | (e-mail* or email* or "electronic mail*" or e-messag* or emessag*).tw. |
| 42 | (wireless adj (communicat* or device* or technolog*)).tw. |
| 43 | ("virtual session*" or "virtual meeting*" or "web session*" or "web meeting*" or "online session*" or "online meeting*" or "digital session*" or "digital meeting*" or "electronic session*" or "electronic meeting*" or "video session*" or "video meeting*").tw. |
| 44 | ((computer* or electronic* or web* or digital* or technolog* or online or video*) adj3 (application* or program* or educat* or learn* or instruct* or guidance* or guiding or platform* or training* or feedback*)).tw. |
| 45 | ((electronic* or digital* or online) adj2 (report* or document* or file*)).tw. |
| 46 | (e-report* or ereport* or e-questionnaire* or equestionnaire*).tw. |
| 47 | technolog* tool*.tw. |
| 48 | ((digital or electronic or online or technologic*) adj system*).tw. |
| 49 | (“computer-mediated communication*” or “computer-assisted instruction*”).tw. |
| 50 | 5 or 6 or 7 or 8 or 9 or 10 or 11 or 12 or 13 or 14 or 15 or 16 or 17 or 18 or 19 or 20 or 21 or 22 or 23 or 24 or 25 or 26 or 27 or 28 or 29 or 30 or 31 or 32 or 33 or 34 or 35 or 36 or 37 or 38 or 39 or 40 or 41 or 42 or 43 or 44 or 45 or 46 or 47 or 48 or 49 |
| 51 | mentor/ |
| 52 | Apprenticeship/ |
| 53 | Professional supervision/ |
| 54 | practicum supervision/ |
| 55 | coaching/ |
| 56 | (student* adj3 (practic* or practis* or placement* or clerkship* or apprentice* or guide or guidance or guiding or train* or instruct* or supervis*)).tw. |
| 57 | (clinic* adj3 (practic* or practis* or placement* or clerkship* or apprentice* or setting* or pedagog* or guide or guidance or guiding or coach* or teach* or learn* or educat* or train* or instruct* or supervis*)).tw. |
| 58 | ((practic* or practis*) adj3 placement*).tw. |
| 59 | (preceptor* or mentor* or practicum*).tw. |
| 60 | (supervis* adj3 (model or models or practic* or practis*)).tw. |
| 61 | training support.tw. |
| 62 | (“clinical field work” or “clinical fieldwork” or “clinical field training”).tw. |
| 63 | 51 or 52 or 53 or 54 or 55 or 56 or 57 or 58 or 59 or 60 or 61 or 62 |
| 64 | 4 and 50 and 63 |
| 65 | limit 64 to ((danish or english or norwegian or portuguese or spanish or swedish) and yr="2011 -Current") |
