# Supplementary material for: Technology-Supported Guidance Models to Stimulate Nursing Students’ Self-Efficacy in Clinical Practice: Scoping Review
Source: JMIR Nurs. 2024 Mar 8;7:e54443. doi: 10.2196/54443 (PMC10960213; doi:10.2196/54443)
Supplement: Multimedia Appendix 2 [file nursing_v7i1e54443_app2.docx]

| **Author, year, country** | **Interventions/ Users’ participation in the intervention development** | **Duration** | **Frequency of usage** |
| --- | --- | --- | --- |
| Chang et al, 2022 [34]  Taiwan | In the first week the participants received an introduction to fundamental knowledge about vaccines for pregnant individuals and pretest. In the second week they utilized the @taiwancdc natural language system to engage in interactive chatting and pose questions, facilitating the acquisition of desired professional knowledge. The participants had the flexibility to express queries or thoughts through the system, utilize a pop-up menu for information searches, and engage in discussions on medical issues with the chatbot. A posttest was conducted at the conclusion of the intervention/ No reported (NR). | 2 weeks | As often as the participants wanted |
| Egilsdottir et al, 2023 [39]  Norway | The participants received oral and written information about the study and completed a pretest at baseline. Over the 8 weeks, participants used the Suite of Mobile Learning Tools, which contains a thoughtful selection of personalized digital learning resources designed to support the learning and application of Basic Physical Assessment Skills (B-PAS). A posttest was performed at the end of the intervention/ The Suite of Mobile Learning Tools was co-designed with students. | 8 weeks | As often as the participants wanted |
| Kim & Suh, 2018 [41]  Korea | The participants completed a pretest at baseline. During the intervention, they used an interactive nursing skills mobile application (ICNS app). The ICNS app was based on the Attention, Relevance, Confidence, Satisfaction (ARCS) theory and the concepts of 3-D nursing simulation education, including videos demonstrating nursing skills such as vital sign measurement, IV injection, gastric gavage, and endotracheal suction. The ICNS app contained interactive learning, emphasizing important techniques through subtitles and pop-up messages. Pop quizzes were also included to reinforce learning. A posttest was performed at the end of the intervention/ NR | 1 week | Not Reported |
| Lee & Park, 2018 [37]  Korea | In the first week before clinical practice, participants completed a pretest and independently studied e-learning materials (consisting of 20 pages of PowerPoint materials and images, along with 10-minute videos) on surgical nursing using the Smart Learning tool. On average, students reported spending 40–50 minutes on pre-learning. Additionally, participants received on-site instruction, providing an opportunity for questions and answers related to the preceding e-learning contents. They also practiced formulating nursing diagnoses based on the knowledge acquired from the e-learning materials. A posttest was conducted at the conclusion of the intervention/ NR. | 45 hours in 1 week | Not Reported |
| Strandell-Laine et al,  2018 [40]  Finland | The participants completed a pretest at baseline. During 5-weeks they engaged in the mobile app Study@CampusPro. The app was designed to facilitate cooperation between student and teacher by placing cooperation procedures in one central, digital environment, allowing flexibility and convenience and continuous use, enabling both synchronous and asynchronous cooperation independent of time and place. The app included the documentation of the schedule of the clinical practicum shifts, learning objectives, a learning diary, mid-term, and final evaluations. The app also included a social networking-style element for individual or group communication between students, teacher, and mentors. A posttest was performed at the end of intervention/ NR. | 5 weeks | Not Reported |
| Wang et al, 2022  [38] China | For 8 weeks the participants used the mobile phone-based psychological intervention program, comprising 3 modules (support, education, and reflection). In the Support module the participants were asked to write a paragraph describing their “happy experiences” during their clinical practice sessions. In the education module, 2 clinical educators provided weekly lectures (1.5 h duration; 4 pm–5:30 pm every Monday) on topics related to nursing knowledge and skills for 8 weeks, and after each lecture the students were required to submit their psychological impression of the lecture. In the reflection module, participants were encouraged to describe the stressful situations encountered during their clinical practice to their clinical educators. A posttest was performed at the end and 6 months after the intervention/ NR. | 8 weeks | Daily |
| Wang et al, 2023 [36]  China | For a duration of 5 weeks, participants engaged in the professional identity program, comprising five weekly sessions led by an instructor. Each session lasted approximately 1.5 hours. The instructor initiated each session with a video greeting to foster familiarity, followed by a review of the previous session and an explanation of the day's activities. Experience-sharing lectures from invited guests occurred in Sessions 2 and 4, while participants shared their homework in Sessions 1, 3, and 5 by opening their screen windows. Subsequently, the instructor encouraged participants to ask questions and express opinions interactively. The session concluded with the dissemination of homework. A posttest was conducted at the conclusion of the intervention/ RN. | 5 weeks | Daily |
| Wu & Sung,  2014 [35]  Taiwan | The participants completed a pretest at baseline. Before the intervention the participants received explanation about how to use Google+ and they were allowed to practice with it. During their practice in home care the participants could share, save, and discuss relevant information on Google+ using any device. Through smart mobile phones, the students could immediately check information, position their locations, upload data, pose questions, and discuss related information. Using the platform, the nurse educator could control the practice of students in different groups and offer feedback and assistance at the appropriate time. A posttest was performed at the end of intervention/ RN. | 4 weeks | Daily |
